# Mind Blindness and Visual Memories

**Published:** 1915-12-11

**Authors:** 


### MIND BLINDNESS AND VISUAL MEMORIES.

### How the Brain Works: the Exceptional Child.

Successful vision as a function of bodily activity involves something more than mere seeing. It involves also, at least on the majority of occasions, identification or recognition. What is desired is not merely the ability to receive visual impressions of various objects in the external world, but, in addition, the ability to interpret these impressions. We must both see and know what we see. Obviously this means that a complete visual apparatus must include an arrangement by means of which visual impressions can be retained or memorised, for identification implies the comparison of the present experience with a former experience of the same order. Without such an arrangement objects might be seen, but there would be no knowledge that they had ever been seen before, and each visual impression would be a new introduction to the object observed. An identical experience might be repeated again and again, but each occasion would be as a new event, for there would be no retained former experience by which the present one could be judged. Objects might be seen but they would not be known. In other words, in order that vision may be of full utility visual impressions of the moment must revive similar impressions received on former occasions, and therefore these latter must be retained in the memory. If by some mischance this store of visual memories is destroyed, the rest of the visual apparatus remaining intact, the person so affected would still be able to see, but the objects seen would be meaningless to him. He would see, but would not understand.

### Mind Blindness.

It is this condition which is known as "mind blindness." The term, of course, affords a contrast to what may be termed physical blindness. Let the eyeballs be seriously damaged by injury or disease, or the lens of each globe be rendered opaque by cataract, and there is necessarily blindness, because rays of light cannot reach the retina, which is the one area on the surface of the body where light can produce nerve activity. The same result, of course, follows disease or destruction of the retina, or of the optic nerve, by which messages excited by retinal activity are conveyed to the visual centres of the brain. Similarly, destruction of the visual centres themselves will produce blindness, for, though the messages reach the brain, there will be no centres to receive them or to make the individual conscious of their reception. In all these instances there will be blindness in the sense that there will be absence of visual sensations. But with eyeballs, optic nerves, and visual centres intact, the physiological conditions for visual sensation are satisfied, and any person possessing these will, therefore, see the objects which are present in the field of vision in front of his eyeballs. If, in addition, he has seen these objects before, and if the part of his brain in which memories of them are stored is in a normal condition, he will not only see the objects, he will also identify them. Should, however, this part of his brain be diseased or destroyed, though he will see the objects quite plainly, they will have no meaning for him. It will be as though he had never seen them before. He will be the victim of mind blindness. In a word, physical blindness may be defined as inability to experience visual sensations, mind blindness as inability to interpret visual sensations.

### Various Visual Memories.

Now, obviously, as there are many kinds or groups of visual sensations, and as the interpretation of each of these depends upon comparison of a present experience with past experiences of the same order, there must be many kinds or groups of visual memories. Impressions made by places, persons, colours, figures, letters, words, etc., must all be accumulated and retained, if, when we see these things again, we are to be able to recognise and interpret them. There are good reasons for believing that these various kinds of visual memories are stored, as it were, not in one and the same region of the brain but in different regions, that, in other words, each group of visual memories is associated with the activity of a special group of nerve centres. Hence it is that mind blindness may be of many different forms, according to the particular group of centres which has been thrown out of action. If, for example, the centres associated with the visual memories of words be diseased, while those associated with other visual memories remain healthy, the person will be mind blind in respect to words, while his visual activities in other respects will be unaffected. Though he sees and interprets other objects, he will see words without being able to interpret them. In similar fashion mind blindness in reference to letters, figures, places, etc., may be present, the corresponding defect, indicated by the name, existing, though in other respects the visual functions are normal. There have even been instances in which mind blindness has existed in reference to the words of one language acquired by the patient, while recognition and interpretation of the words of another language have been retained. Thus there is much evidence to show that the visual memories upon which the interpretation of visual impressions depends have an anatomical basis in the brain, and that each group is related to a particular brain area. When this is accepted the different forms of mind blindness readily find their explanation in the localisation of brain disease to one or other of the corresponding districts.

The most confident knowledge relative to the localisation of visual memories is concerned with the visual memory for words and letters. It is known that word blindness and letter blindness are results of disease affecting the left side of the brain in the hinder portion of the parietal lobe, and these are the forms of mind blindness which have most frequently been known and studied, though, as indicated above, several other varieties have been recognised. So certain is the association between word blindness and the area just mentioned that the occurrence of the defect may be accepted as proof of the exact site of the brain lesion. In the great majority of cases of brain disease the mischief is not severely restricted to a limited district, and hence many functions other than that of the word-memory centres are likely to be disturbed, even when these centres themselves are actually involved. Hence there are cases in which it may be difficult or impossible to recognise that there is loss of the visual memories for words. A patient, for example, from disease in his left brain, may completely lose the power of speech, and in such circumstances it may not be easy to isolate the failure of his visual word memories. But examples do occur of what may be called pure mind blindness in relation to words, where the patient, though conscious of no other defect, finds himself unable to read while quite able to see clearly the printed or written characters.

### The Hinshelwood Case.

One of the most striking instances of this condition was reported some years ago by Dr. James Hinshelwood, who has made a special and original study of the whole subject. The patient, a man of fifty-eight years, was a teacher of French and German, who suddenly found himself unable to read the exercises given to him by his pupils. Taking up a printed book, he could not recognise a single word; the page appeared to him just as it appears to a person who has never learned to read. Yet with this there was no interference with his general intelligence or memory, and but for the one defect he regarded himself as in his usual health. A very interesting feature in this case was the fact that with this striking deficiency in respect to words and letters the patient had no difficulty at all with figures. He had lost his visual memories of words, but retained his visual memories of figures, an observation which seems to compel the conclusion that the two groups of memories are located in different though perhaps neighbouring brain areas. The experience is a most valuable one in regard both to the physiological working of the visual apparatus and also in reference to the localisation of brain disease. There were certain other technical reasons for concluding that the disease in this instance was limited to the left side, and manifestly it was of very restricted distribution. Altogether there can be no doubt that here was an instance in which, while the seeing or perceiving part of the mechanism for vision remained intact, the interpreting function, so far as words and letters are concerned, failed as a result of a localised brain lesion which destroyed the centres where the visual memories for words and letters are normally retained. Another interesting feature in Dr. Hinshelwood's case is that the patient, after some months of precautionary rest, took up the task of learning to read, commencing with the letters of the alphabet and a child's primer after the fashion pursued in an infant school and he obtained a considerable measure of success. He had to provide himself with a new set of visual word memories, and presumably he did this by the education of a new area of brain substance.

### The Exceptional Child.

There is another aspect of the subject which has some importance to those concerned with education, and, in particular, perhaps, to doctors occupied with the medical inspection of school children. Every now and again a school teacher discovers a child who has quite exceptional difficulty in learning to read and spell. In other respects the child may appear quite normal, and the temptation is to conclude that the deficiency is due to laziness or carelessness or obstinacy. There is, however, good reason to believe that there is such a condition as congenital word blindness, due, presumably, to some defect—quantitative or qualitative—in the brain area whose proper function is the accumulation of visual word memories. To label these children, or at all events all of them, as mentally defective seems to be in excess of the facts, and yet without doing so it may be difficult to obtain for them the special training they need. Thus mind blindness touches many practical areas, and in one of its relations has an important association with the work of our elementary schools. The subject was recently discussed at one of the medical societies, and Dr. Shuttleworth, who speaks with considerable authority on any question relating to congenital mental defects, expressed the opinion that congenital word and letter blindness was, at least in some degree, much more frequent than is generally believed. And, after all, this is not so very surprising in view of the notorious differences of mental capacity in children in regard both to the ease with which they acquire knowledge and to the firmness with which they retain it.

It is to be noted that to speak of "memory" as a single capacity or function is probably a mistake. What is true of visual impressions is true also of other sensations. It is possible to produce physical deafness by methods comparable to those which cause physical blindness; and equally, by destruction of certain brain centres, it is possible to cause mind deafness in relation, say, to words, where the person would hear the sound of words but would have lost the power to understand their meaning in consequence of the destruction of his store of auditory word-memories. That is to say, that the physiological arrangements provide a basis not for *a* memory but for memories, and hence it is readily possible to have acuteness in one group and not in another. Common experience, too, seems to confirm this, for it is notorious that some individuals remember with great success things they have seen (visual memories) while their memory capacity for spoken words is very poor (auditory memories); or the arrangement may be the other way about. The bearing of this on the discussion of any child's "mental deficiency" and on the method of his education is obvious.

